# ASSESSING FALLS RISK AND BALANCE SYSTEMS PROTOCOLS IN ADULT CANCER SURVIVORS

**DOI:** 10.1093/geroni/igae098.2250

**Published:** 2024-12-31

**Authors:** Kenzi Marrone-Lloyd, Kelli Nielsen, Emma Christopher, Raegan Hinrichs, Caitlin O’Donnell, Laura Gilchrist

**Affiliations:** St. Catherine University, Saint Paul, Minnesota, United States; St. Catherine University, Saint Paul, Minnesota, United States; St. Catherine University, Saint Paul, Minnesota, United States; St. Catherine University, Saint Paul, Minnesota, United States; St. Catherine University, Saint Paul, Minnesota, United States; St. Catherine University, Saint Paul, Minnesota, United States

## Abstract

Adult cancer survivors have been shown to have an elevated risk of falling compared to control. These risks are hypothesized to be residual effects of chemotherapy treatment that can impact patients’ balance long-term through changes to vestibular, visual, and somatosensation systems. Medical providers and rehabilitation professionals may be asked to screen for fall risk, yet best screening measures for these balance system changes have not been identified. The purpose of this study is to determine if balance assessment outcomes differ between adult cancer survivors and control groups and if these balance outcomes are associated with prior falls history. Twenty participants underwent a series of sensory organization testing (SOT) using computerized posturography (BERTEC) and the MiniBest Test, which measures dynamic balance, gait and functional mobility. Cancer survivors (n=10) fell more than their control (n=10) counterparts (X²=3.81, p=0.050). On the SOT, cancer survivors (88.2 ± 13.4) had a preference for vision, even when distorted, compared to control (100.3 ± 11.9) (t=2.14, p=.023) however, scores for all other tests were not different between groups (p>0.05). This preliminary data supports that cancer survivors fall more often than non-cancer survivors and that cancer survivors rely more on vision, as a means to maintain balance. Indicating that other balance systems may be unreliable or damaged. Continued development of screening tools to gauge the impact of cancer on balance and fall risk is indicated to improve the care of cancer survivors.

